# Repeated injections of vitamin E and Se improves testicular morphology, testosterone and in vitro and in vivo sperm fertility in subfertile rabbits

**DOI:** 10.1007/s11259-024-10439-4

**Published:** 2024-08-07

**Authors:** Aya M. Fadl, Haney Samir, Abdallah M. Shahat

**Affiliations:** 1 Department of Theriogenology, Faculty of Veterinary Medicine, Cairo University, Giza, Egypt; 2 Laboratory of Veterinary Physiology, Department of Veterinary Medicine, Faculty of Agriculture, Tokyo University of Agriculture and Technology, 3-5-8 Saiwai-Cho, Fuchu, 183-8509 Tokyo, Japan

**Keywords:** Fertility, Freezability, Rabbits, Selenium, Subfertility, Vitamin E

## Abstract

Subfertility is a multifactorial disorder that affects the rabbit production industry. However, subfertility may be treated by using a simple intervention such as vitamin supplementation. Vitamin E and selenium (Se) are potent antioxidants that protect the male reproductive system. The aim of this study is to determine the effects of vitamin E and Se on testicular size, semen quality and freezability, antioxidant activity, testosterone levels, and fertility in subfertile rabbits. Twenty-one New Zealand rabbits were classified as subfertile rabbits based on their semen characteristics and fertility records. The rabbits were randomly allocated into 3 equal groups (G1: control; G2: injected with Vit E 100 IU/head + Se 0.1 mg/kg b.w.; G3: injected with Vit E 200 IU/head + Se 0.2 mg/kg b.w. once weekly for 8 weeks).Once weekly for 8 W, blood samples were collected to measure serum testosterone level and total antioxidant capacity (TAC), and semen samples were collected by artificial vagina to assess the quality of fresh and frozen semen. At the 8th week of the study, 150 multiparous does were artificially inseminated with fresh semen to assess the fertility of rabbits after treatment; 50 does for each group. At the end of the study, rabbits were slaughtered to assess testicular morphometry. Fresh and post-thaw semen quality parameters were significantly (*p* < 0.05) higher in G3in comparison with G2and G1, respectively. Also, testosterone level was significantly (*p* < 0.05) increased at the 2nd week in G3in comparison with other groups. Conception and kindling rates were significantly (*p* < 0.05) higher in does which were inseminated with semen fromG3. In conclusion, injection of vitamin E and selenium at a higher dose (G3) improved the testicular morphology, quality of fresh and post-thaw semen, and most importantly, the fertility of subfertile rabbits.

## Introduction

Rabbits have a high reproductive rate compared to other livestock. In addition to being an excellent source of dietary protein for human nutrition, rabbits are an excellent model for the different aspects of research in reproduction (Fischer et al. 2012).

Fertility is the ability to produce offspring, while infertility is the inability to reproduce. Furthermore, subfertility is a complex condition (environmental, lifestyle, and genetic) that refers to the failure of conception for a long period of time. In the literature and clinical contexts, this phrase is frequently used wrongly as synonymous with infertility. However, subfertility may be treated by using a simple intervention such as vitamin supplementation (Salma et al. 2011).

Oxidative stress (OS) is an arising factor in unexplained male fertility problems (Sharlip et al. 2002). Furthermore, OS is characterized by excessive generation and accumulation of reactive oxygen species (ROS), which is accompanied by the decrement and weakness of the endogenous antioxidant system leading to oxidative damage of sperm proteins, lipids, and nucleic acids (Mannucci et al. 2022).

Rabbit sperm plasma membrane is highly vulnerable to free radicals induced lipid peroxidation due to containing high levels of polyunsaturated fatty acids (PUFAs) and low antioxidant levels within the cytoplasm (El-Gendy 2022).

Accordingly, many strategies can be used to mitigate the adverse effects of oxidative stress on reproduction using vitamins, minerals, and/or elements with antioxidant potential (Akarsu et al. 2023a, 2023b) thereby enhancing fertility potential (Brecchia et al. 2023).

Vitamin E (vit E) is a major fat-soluble antioxidant vitamin present in cell membranes that mitigates oxidative damage by breaking the chain reaction of peroxidation and inhibiting the generation of ROS. Furthermore, many previous studies revealed that the deficiency of vit E could adversely affect fertility potential (Wang et al. 2022). Moreover, it has the potential to neutralize free radicals, thereby protecting cell membranes from them (Kessopoulou et al. 1995).

Selenium (Se) is an important trace element responsible for various reproductive functions, including testicular development, spermatogenesis, and testosterone metabolism (Moslemi and Tavanbakhsh 2011). In addition, Se is a critical constituent of sperm capsule selenoproteins that protect the membrane from peroxidation by ROS and increase the activity of GSH-peroxidase (Burk and Hill 2009). Moreover, it is mostly found in the active sites of enzymes in the form of selenocysteine. Multiple Se-containing proteins, including GSH-Px and thioredoxin reductase, play critical roles in avoiding oxidative damage (Ventura et al. 2017). As a result, the usefulness of selenium supplementation in increasing internal antioxidant defense has been highlighted in recent years. Several previous studies were conducted on rabbits (Hosny et al. 2020)to study the effect of Vit E and se on the reproductive performance. On the other hand, no previous reports were conducted to investigate vit E and Se injection on the semen quality and fertility of subfertile rabbits. The aim of this study was to investigate the effect of repeated injection of vit E and Se on the quality of semen (fresh and frozen), testicular morphometry, testosterone, and total antioxidants capacity (TAC) levels, and the overall reproductive performance of the subfertile rabbits. We hypothesized that injection of vit E and Se once weekly for 8 W may improve the quality, preservability, and fertility potential of the semen, thereby enhancing testosterone levels and the antioxidant status of subfertile rabbits.

## Materials and methods

Unless otherwise stated, all chemicals were purchased from Sigma-Aldrich (Madrid, Spain). The current investigation was conducted fromSeptember 2022 to April 2023 at Theriogenology Department Farm, Faculty of Veterinary Medicine, Cairo University, Egypt. All experimental procedures were reviewed and approved by the Ethical Committee for Animal use belonging to the Faculty of Veterinary Medicine, Cairo University, Egypt (Vet CU 03162023751).

### Animals

Two hundred and forty-three rabbits were screened out using semen evaluation and fertility records to keep twenty-one adult New Zealand rabbits aged18 months, weighing 5–6 kg, for use in the present study. Rabbits had a history of decreasing fertility (low conception rate ˂ 30% after natural mating with fertile multiparous does for several times) and low semen quality (motility, viability percentages were < 60% and sperm cell concentrations were < 250 ˟ 10^6^/ml; Fadl et al. 2019). Based on fertility records and rabbits’ semen characteristics, rabbits were identified as subfertile. Rabbits were individually housed in wire cages and fed a standard commercial diet (crude fibre 19%, crude protein 18%, fat 3%, phosphorus 0.5%, and calcium 1.00%) following the NRC guidelines (NRC 1977) and managed in a natural environment (temperatures ranged from 19 °C to 25 °C). Freshwater was freely accessible *ad libitum*. Rabbits were allotted into three groups: a control group (G1, *n* = 7) that received no antioxidant treatment (only saline) and two treatment groups (G2 & G3). G2 (*n* = 7) was injected intramuscularly with Vit E (100 IU/head) and Se (0.1 mg/kg b.w.), while G3 (*n* = 7) was injected with Vit E (200 IU/head) and Se (0.2 mg/kg b.w.). The treatment protocol for the studied rabbits was once weekly for eight successive weeks based on the duration of spermatogenesis in rabbits (about 53 d) (Swierstra and Foote 1965). The dose of Vit E and Se (Cairo Company for Medicine, Egypt) was selected based on a previous report (Meshreky and Metry 2000).

### Experimental design

#### Experiment 1: Effects of repeated injections of Vit E and Se on the quality of fresh and frozen/thawed subfertile rabbit semen

Once per week for 8 consecutive weeks (from W1 to W8), semen was collected from rabbits using prewarmed (42ºC) and lubricated artificial vagina specified for rabbits. Immediately after semen collection, the gel portion was separated, and ejaculates were incubated at 37 ºC for sperm quality parameters (volume, concentration, motility, viability, and acrosome integrity) assessment. Collected ejaculates for each group were pooled to avoid individual variation between rabbits, and then pooled semen was evaluated.

For cryopreservation, pooled semen for each group was diluted (1:1) with INRA-82 extender (Vidament et al. 2000) and fortified with dimethyl formamide 4%+ dimethyl sulfoxide 4% as cryoprotectants (Fadl et al. 2019). Then, diluted semen samples were kept in the refrigerator at 5 °C for cooling (for 90 min) and equilibration (for 15 min). After equilibration, cooled semen was loaded into 0.5 mL plastic straws and sealed with polyvinyl powder. Straws were placed horizontally at 4 cm over liquid nitrogen (LN₂) vapours for 10 min and plunged into LN₂ for storage. For evaluation of post-thaw semen parameters (motility, viability, and acrosome integrity), straws (after at least one week in storage) were thawed using a water bath at 50 °C for 7 s (Di Iorio 2014).

#### Fresh and post-thaw semen evaluation

#### Volume and concentration

The ejaculate volume was assessed using a graduated tube. Sperm cell concentration was evaluated by the direct sperm cell count method using the Neubauer haemocytometer (Number of sperm counted* Dilution factor* 50,000 sperm/ml), according to Smith and Mayer (1955).

#### Individual motility

Individual motility was evaluated in fresh and post-thaw semen samples. A drop of semen was placed on a preheated 37 °C glass slide, covered with a cover slide (37 °C), and then examined under an optical microscope (Olympus® BH-2, made in Japan) with an adjusted hot stage at 38–40 °C. Semen samples were subjectively assessed for sperm motility by the same person after examination of several microscopic fields and recorded in 5% increments (range 0-100%) according to the method previously described by Evans and Maxwell (1987).

#### Viability and morphology

The viability and normal sperm morphology were evaluated using eosin-nigrosin stain, as mentioned previously by Evans and Maxwell (1987). In detail, a drop of fresh or post-thaw semen was mixed with a drop of the stain and smeared on a pre-warmed (37 °C) glass slide, left to dry, and then examined microscopically using a bright field microscope (1000×) to assess the viability and sperm morphology of a total of 200 spermatozoa. On the same slide used for viability assessment, the abnormal morphology of spermatozoa was recorded.

#### Acrosome integrity

Acrosome integrity was evaluated in fresh and frozen-thawed semen using Spermac stain (FertiPro N.V., Belgium) as previously described by Chan et al. (1999). Using a bright field microscope, a total of 200 sperm were recorded, and the percentages of intact acrosomes were calculated.

### Experiment 2: Effects of repeated injections of Vit E and Se on the serum concentration testosterone and total antioxidant capacity of subfertile rabbit

Once per week for 8 consecutive weeks (from W1 to W8) in the morning before access feed, blood samples were obtained from the marginal ear vein of rabbits and placed into plain tubes. The drawn blood samples were subjected to centrifugation at 3000 rpm for 15 min and then the serum samples were collected and stored (− 20 ℃) for hormonal and biochemical analyses (Shetaewi 1998).

Serum testosterone concentration was measured as previously mentioned by Abraham et al. (1977) using commercial kits (Diagnostic Production Corporation, Los Angeles, USA).

Serum total antioxidant capacity (TAC) was determined spectrophotometrically using commercial kits (total antioxidant capacity, Bio-diagnostic, Dokki, Egypt), according to Ippoushi et al. (2005).

### Experiment 3: Effects of repeated injections of Vit E and Se on the fertility of subfertile rabbit semen

One hundred and fifty New Zealand white multiparous does aged 10–12 months and weighing 4–6 kg have been used for evaluation the fertility of subfertile rabbit semen after repeated injections of Vit E and Se for 8 W. They were randomly allotted into three groups (*n* = 50 for each group). Fresh pooled semen for each group was diluted with INRA-82 extender. Dilution rates were calculated according to sperm cell concentration and insemination volume. Based on this, receptive does (vulvae lips have a dark pinkish coloration) were deeply inseminated with 0.5 mL of extended semen containing about 25 × 10^6^ spermatozoa using a curved inseminating pipette (Imporvet, S.A.). Immediately after insemination, does were injected with buserelin acetate (Receptal; Intervet International GmbH Feldstrasse 1a D-85716Unterschleißheim, Germany) in a dose of 0.8 µg (0.2 ml/ doe) for induction of ovulation. On the 15th day after insemination, an ultrasound examination was performed to assess pregnancy and calculate the conception rate (number of pregnant females/number of inseminations). After parturition, kindling rates were calculated (number of does giving birth/number of inseminations).

### Experiment 4: Effects of repeated injections of Vit E and Se on the testicular morphometery of subfertile rabbit

At the end of the study, three rabbits from each treatment were randomly selected and humanely sacrificed. After sacrifice, paired testes for each rabbit were dissected (without epididymis) for morphometric assessment (weight, length, width, and volume). To assess the testicular weight, an electronic scale was used for this purpose. Using a pair of vernier callipers, testicular length, and width were measured. Furthermore, testicular volumes in all bucks were determined by water displacement according to the Archimedes principle, as previously mentioned by Adu and Egbunike (2010).

### Statistical analysis

The normality of data distributions was evaluated, and a two-way ANOVA was used to analyse fresh and frozen semen parameters, testosterone, and TAC levels. While conception and kindling rates and testicular morphometry results were analysed using a one-way ANOVA followed by a Bonferroni post-hoc test. Results are reported as mean ± standard error of the mean (SEM). For all analyses, *P* < 0.05 was considered significant. The IBM SPSS 27.0 Software Package was used to conduct statistical analyses (IBM Corp., New York, NY, USA).

## Results

### Semen parameters

The quality parameters of fresh semen were presented in Table 1. There were effects of treatment, week, and treatment*week interaction effects (*P* < 0.05). Individual motility, viability (W4 to W8), sperm cell concentration (W6 to W8), normal sperm morphology (W7 to W8) and intact acrosomes (W6 to W8) were significantly increased in G3 in comparison with G2 and G1 groups.

### Post-thaw sperm parameters

There were effects of treatment, week and an interaction between treatment and week (*P* < 0.05). As depicted in Table 2, the percentage of post-thaw sperm motility (W3 to W8), viability (W3 to W8), normal sperm morphology (W4 to W8), and acrosome integrity (W4 to W8) was higher in G3 compared to other groups.

### Serum testosterone and total antioxidant capacity (TAC) levels

**Fig. 1:**
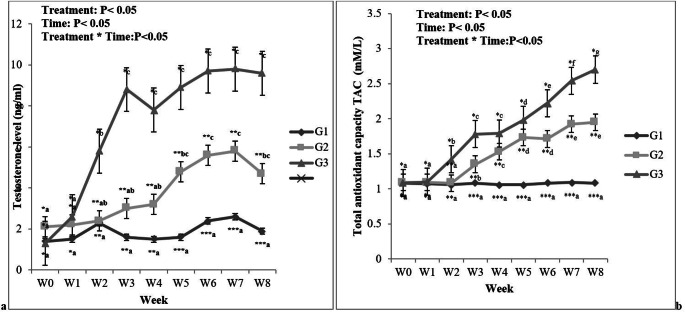
**a**, **b** Effects of VitE and Se injection at different doses on the serum concentrations of testosterone and total antioxidant capacity (TAC). G1: *n *= 7, control group. G2: *n *= 7, rabbit injected with Vit E 100 IU/head +Se 0.1 mg/kg b.w. G3: *n* = 7, rabbit injected with Vit E 200 IU/head +Se 0.2 mg/kg b.w. Values are means ± SEM. ^.*,**,***^Values in each parameter are significantly different at least at *P* < 0.05 between the groups at the indicated time. ^a b,c^Values represent significant (*P* < 0.05) time differences within treatment during the study. Treatment: *P *< 0.05; Time: *P *< 0.05; Treatment * Time: *P *< 0.05

Serum testosterone and TAC levels were presented in Fig. 1a, b. There were effects of treatment, week, and treatment*week interaction effects (*P* < 0.05). Serum testosterone concentrations and TAC levels were significantly increased in G3 from W2 to W8 compared to G1.

### Fertility results

**Fig. 2:**
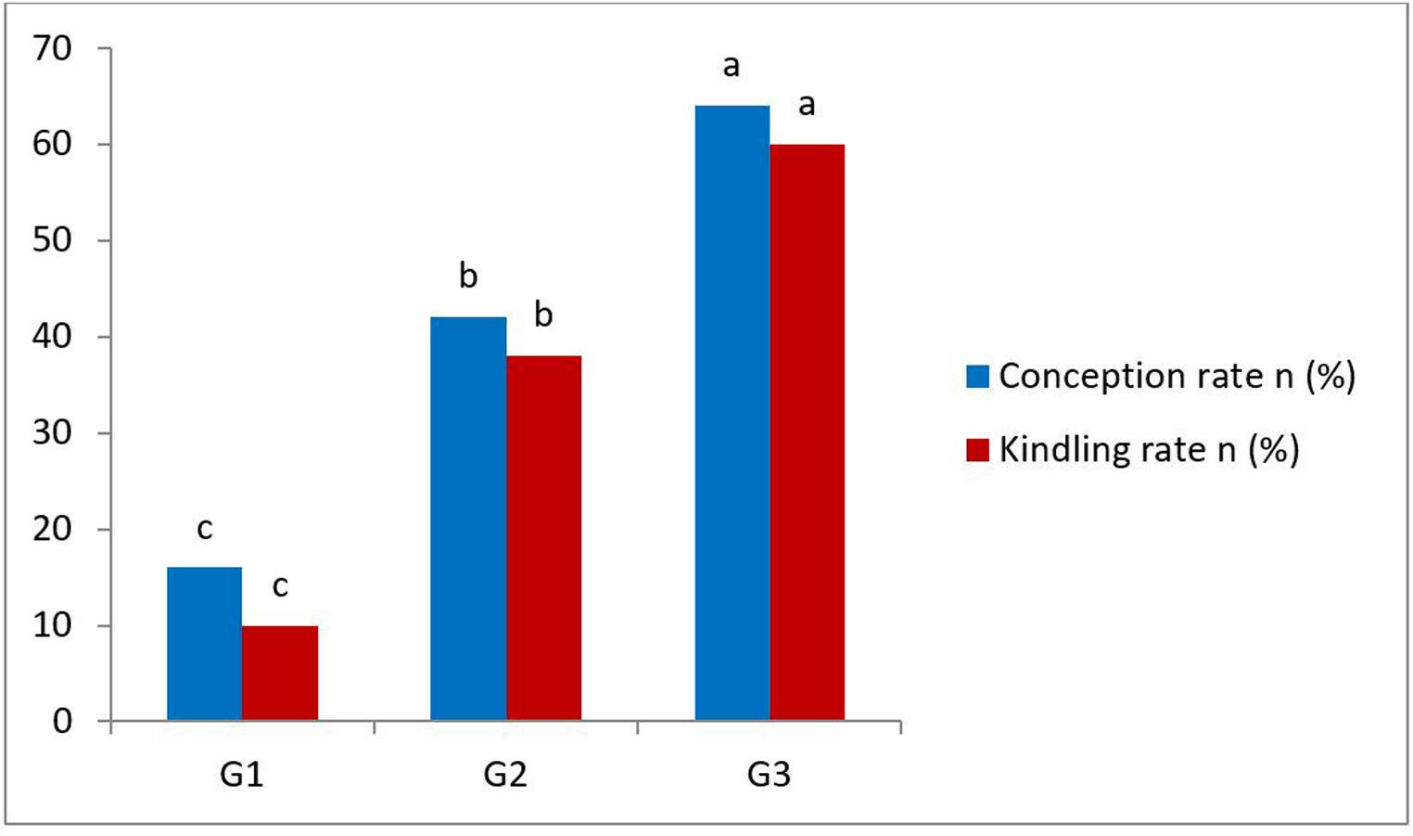
In vivo reproductive performance of rabbit does were inseminated with rabbit semen after injection of Vit E and Se at different doses for 8 weeks. G1:*n *= 50, control group. G2: *n *= 50, rabbit does were inseminated with rabbit semen that injected with Vit E 100 IU/head +Se 0.1 mg/kg b.w. G3: *n* = 50, rabbit does inseminated with rabbit semen that injected with Vit E 200 IU/head +Se 0.2 mg/kg b.w. Values with different superscripts a,b, and c indicate a significant difference (*P* < 0.05)

As depicted in Fig. 2, G3 showed a significant increase in conception and kindling rates in comparison with G2 and G1 groups.

### Testicular morphometry

**Fig. 3:**
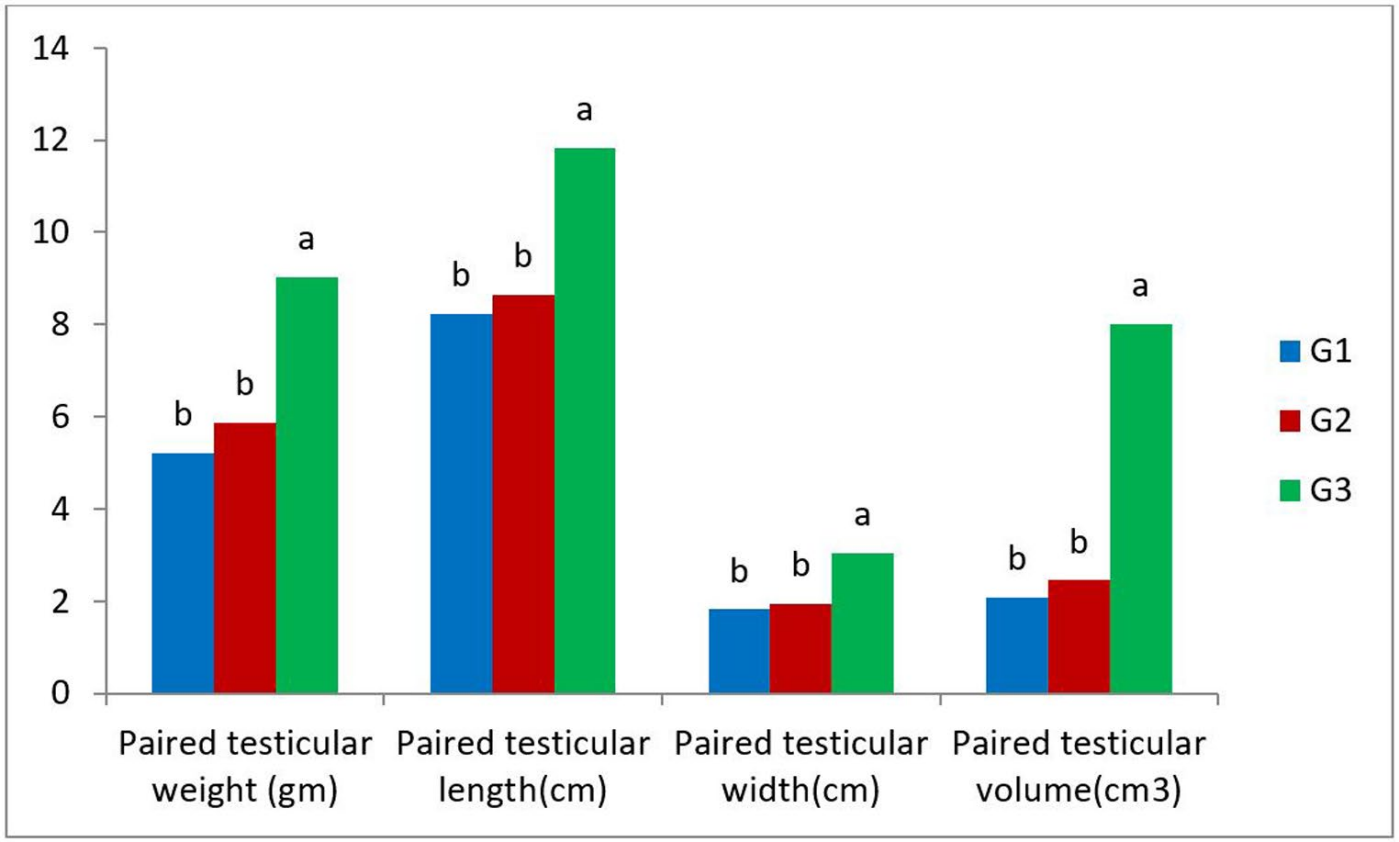
Effects of VitE and Se injection at different doses on the testicular morphometry of rabbits. G1: *n *= 3, control group. G2: *n *= 3, rabbits injected with Vit E 100 IU/head +Se 0.1 mg/kg b.w for 8 weeks. G3: *n* =3, rabbits injected with Vit E 200 IU/head +Se 0.2 mg/kg b.w for 8 weeks. Values in each parameter with different alphabetical superscripts are significantly different at least at *P* < 0.05

Testicular morphometry results are presented in Fig. 3. Values of testicular weight, length, width, and volume were significantly increased in G3 compared to other groups.

**Table 1:** Effect of Vit E and Se injection on the quality of fresh rabbit semen

	Volume (ml)			Sperm cell concentration *10^6^/ml			Individual motility (%)			Viability (%)			Normal morphology (%)			Intact acrosomes (%)		
	G1	G2	G3	G1	G2	G3	G1	G2	G3	G1	G2	G3	G1	G2	G3	G1	G2	G3
W0	0.80 ± 0.21	0.76 ± 0.32	0.79 ± 0.45	212.12 ± 0.32	222.23 ± 0.18	216.45 ± 0.32	49.16 ± 0.23	48.33 ± 0.12	47.03 ± 0.67	54.23 ± 0.17	54.76 ± 0.52	53.12 ± 0.33	70.34 ± 0.82	71.21 ± 0.13	70.44 ± 0.22	75.23 ± 0.08	73.62 ± 0.37	75.88 ± 0.28
W1	0.72 ± 0.24	0.91 ± 0.12	0.83 ± 0.51	219.34 ± 0.11	217.33 ± 0.42	227.12 ± 0.21	51.23 ± 0.34	51.12 ± 0.38	50.47 ± 0.46	55.12 ± 0.36	54.88 ± 0.39	55.34 ± 0.72	68.72 ± 0.61	73.02 ± 0.43	69.53 ± 0.64	73.38 ± 0.14	74.13 ± 0.61	73.14 ± 0.32
W2	0.86 ± 0.13	0.88 ± 0.25	0.77 ± 0.41	210.14 ± 0.54	228.24 ± 0.13	225.83 ± 0.27	48.41 ± 0.61	48.24 ± 0.28	51.32 ± 0.18	52.31 ± 0.12	53.18 ± 0.22	56.12 ± 0.68	71.48 ± 0.53	70.88 ± 0.71	72.84 ± 0.18	76.18 ± 0.23	74.68 ± 0.46	77.22 ± 0.16
W3	0.84 ± 0.32	0.82 ± 0.22	0.88 ± 0.36	220.17 ± 0.48	225.32 ± 0.52	232.71 ± 0.15	48.18 ± 0.32	50.12 ± 0.21	49.24 ± 0.22	53.46 ± 0.42	55.26 ± 0.18	53.93 ± 0.25	69.66 ± 0.32	71.86 ± 0.14	73.31 ± 0.43	75.78 ± 0.52	76.42 ± 0.28	75.43 ± 0.41
W4	0.78 ± 0.28	0.88 ± 0.18	0.79 ± 0.42	221.11 ± 0.61	236.51 ± 0.38	238.16 ± 0.34	50.64 ± 0.72^**a^	51.22 ± 0.35^**a^	59.67 ± 0.38^*b^	54.82 ± 0.27^**a^	55.49 ± 0.41^**a^	64.16 ± 0.31^*b^	70.25 ± 0.13	72.29 ± 0.23	73.48 ± 0.12	74.30 ± 0.65	73.82 ± 0.37	77.63 ± 0.56
W5	0.71 ± 0.35	0.78 ± 0.25	0.85 ± 0.29	216.42 ± 0.26	240.11 ± 0.46	242.41 ± 0.56	49.36 ± 1.41^**a^	52.18 ± 1.29^**a^	63.52 ± 1.41^*b^	54.66 ± 1.13^**a^	58.02 ± 1.34^**a^	69.14 ± 1.16^*b^	71.41 ± 1.57	73.22 ± 1.91	75.12 ± 1.62	77.03 ± 1.12	76.12 ± 1.31	79.01 ± 1.14
W6	0.87 ± 0.14	0.89 ± 0.14	0.82 ± 0.45	226.53 ± 0.48^**a^	246.53 ± 0.19^**a^	288.23 ± 0.38^*b^	51.12 ± 0.13^***a^	61.14 ± 0.11^**b^	74.47 ± 0.21^*c^	55.46 ± 0.10^***a^	65.34 ± 0.11^**b^	76.13 ± 0.26^*c^	68.72 ± 0.28	72.30 ± 0.47	74.18 ± 0.41	75.55 ± 0.10^**a^	77.17 ± 0.28^**a^	87.11 ± 0.34^*b^
W7	0.79 ± 0.23	0.77 ± 00.24	0.79 ± 0.29	213.24 ± 0.37^***a^	289.23 ± 0.35^**b^	340.54 ± 0.49^*c^	48.62 ± 0.82^***a^	63.32 ± 0.24^**b^	74.53 ± 0.33^*c^	53.62 ± 0.19^***a^	67.15 ± 0.31^**b^	81.28 ± 0.23^*c^	69.47 ± 0.64^**a^	74.52 ± 0.15^**a^	82.22 ± 0.52^*b^	74.34 ± 0.28^**a^	76.81 ± 0.51^**a^	88.13 ± 0.22^*b^
W8	0.81 ± 0.22	0.85 ± 00.19	0.82 ± 0.31	210.61 ± 0.29^***a^	312.45 ± 0.21^**b^	356.67 ± 0.32^*c^	51.38 ± 0.22^***a^	62.31 ± 0.29^**b^	75.18 ± 0.19^*c^	54.89 ± 0.38^***a^	68.28 ± 0.62^**b^	81.34 ± 0.31^*c^	70.21 ± 0.34^**a^	74.87 ± 0.20^**a^	85.13 ± 0.62^*b^	73.96 ± 0.37^**a^	77.72 ± 0.36^**a^	88.24 ± 0.31^*b^

G1: *n *= 7, control group

G2: *n *= 7, rabbitsinjected with Vit E 100 IU/head +Se 0.1 mg/kg b.w

G3: *n* = 7, rabbitsinjected with Vit E 200 IU/head +Se 0.2 mg/kg b.w

Values are means ± SEM

^.*,**,***^Values in each parameter are significantly different at least at *P* < 0.05 between the groups at the indicated time

^a b,c^Values represent significant (*P* < 0.05) time differences within treatment during the study

Values in each parameters haven’t superscripts aren’t significantly different  at *P* < 0.05

Treatment: *P *< 0.05;Time: *P *< 0.05; Treatment * Time: *P *< 0.05

**Table 2:** Effect of Vit E and Se injection on the quality of post-thaw rabbit semen

	Individual motility (%)			Viability (%)			Normal morphology (%)			Intact acrosomes (%)		
	G1	G2	G3	G1	G2	G3	G1	G2	G3	G1	G2	G3
W0	11.12 ± 0.35	10.23 ± 0.26	10.44 ± 0.39	16.35 ± 0.12	15.33 ± 0.72	16.23 ± 0.09	45.42 ± 0.27	47.28 ± 0.63	47.66 ± 0.34	56.14 ± 0.25	55.31 ± 0.53	53.82 ± 0.26
W1	15.47 ± 0.22	14.49 ± 0.42	13.07 ± 0.27	19.41 ± 0.63	18.91 ± 0.54	19.34 ± 0.24	45.84 ± 0.12	45.12 ± 0.23	44.36 ± 0.40	54.12 ± 0.37	54.23 ± 0.42	55.15 ± 0.34
W2	11.42 ± 0.38	12.38 ± 0.31	15.12 ± 0.34	16.27 ± 0.55	17.39 ± 0.28	20.12 ± 0.11	47.32 ± 0.24	46.88 ± 0.91	46.50 ± 0.54	54.32 ± 0.16	56.18 ± 0.13	54.71 ± 0.24
W3	12.33 ± 0.10^**a^	14.51 ± 0.37^**a^	26.29 ± 0.17^*b^	17.43 ± 0.21^**a^	19.21 ± 0.51^**b^	31.36 ± 0.45^*b^	46.38 ± 0.39	44.26 ± 0.14	46.61 ± 0.28	55.82 ± 0.41	55.64 ± 0.81	57.68 ± 0.13
W4	15.62 ± 0.41^***a^	22.13 ± 0.23^**b^	30.54 ± 0.38^*bc^	20.11 ± 0.35^***a^	28.42 ± 0.32^**c^	36.07 ± 0.26^*bc^	45.31 ± 0.45^**a^	47.08 ± 0.18^**a^	55.57 ± 0.25^*b^	56.21 ± 0.32^**a^	57.21 ± 0.33^**a^	66.14 ± 0.26^*b^
W5	13.44 ± 0.18^***a^	26.45 ± 0.15^**bc^	33.67 ± 0.12^*c^	18.82 ± 0.29^***a^	30.25 ± 0.41^**cd^	38.67 ± 0.22^*c^	45.79 ± 0.51^**a^	46.16 ± 0.31^**a^	55.23 ± 0.38^*b^	55.08 ± 0.43^**a^	58.14 ± 0.54^**a^	74.38 ± 0.53^*c^
W6	15.50 ± 0.35^***a^	30.04 ± 0.23^**c^	42.36 ± 0.31^*d^	19.67 ± 0.33^***a^	35.17 ± 0.12^**d^	47.31 ± 0.37^*d^	46.22 ± 0.16^**a^	47.23 ± 0.53^**a^	56.44 ± 0.14^*b^	56.40 ± 0.63^***a^	65.18 ± 0.47^**b^	76.16 ± 0.29^*c^
W7	12.48 ± 0.63^***a^	38.42 ± 0.16^**d^	45.81 ± 0.10^*d^	18.21 ± 0.41^***a^	44.47 ± 0.33^**e^	52.12 ± 0.55^*de^	45.38 ± 0.32^***a^	54.12 ± 0.34^**b^	65.52 ± 0.17^*c^	54.48 ± 0.37^***a^	66.26 ± 0.36^**b^	75.51 ± 0.23^*c^
W8	15.83 ± 0.24^***a^	38.32 ± 0.26^**d^	47.39 ± 0.24^*d^	20.64 ± 0.32^***a^	43.38 ± 0.27^**e^	53.78 ± 0.14^*e^	46.28 ± 0.52^***a^	55.25 ± 0.41^**b^	65.14 ± 0.45^*c^	56.16 ± 0.42^***a^	66.22 ± 0.48^**b^	76.12 ± 0.31^*c^

G1: *n *= 7, control group

G2: *n *=7, rabbits injected with Vit E 100 IU/head +Se 0.1 mg/kg b.w

G3: *n* =7, rabbits injected with Vit E 200 IU/head +Se 0.2 mg/kg b.w

Values are means ± SEM

^.*,**,***^Values in each parameter are significantly different at least at *P* < 0.05 between the groups at the indicated time

^a b,c^Values represent significant (*P* < 0.05) time differences within treatment during the study

Values in each parameters haven’t superscripts aren’t significantly different at *P* < 0.05 Treatment: *P* < 0.05; Time: *P *< 0.05

Treatment * Time: *P *< 0.05

## Discussion

The objective of the current study was to elucidate whether repeated injection of Vit E and Se affected the sperm quality and fertility of subfertile rabbits. No previous reports investigated the effects of the injection of Vit E and Se on the fresh and preserved semen quality, testosterone and TAC levels, and testicular size of subfertile rabbits. In the present study, treated groups (G3 and G2, respectively) had higher sperm (fresh and preserved) individual motility, viability and testosterone, TAC concentrations, conception, kindling rates, and testicular size compared to the control group (G1). These results supported the hypothesis that the injection of vitamin E and Se positively affects the quality and fertility parameters of fresh and cryopreserved semen from rabbits suffering from subfertility.

Semen quality is a good indicator of fertility (Bas et al. 2023). Rabbit sperm membrane contains high concentrations of polyunsaturated fatty acids, which makes the sperm cell very sensitive to peroxidative damage by ROS (Fadl et al. 2021). For normal sperm function, limited amounts of ROS are necessary. Despite the positive effect of ROS on sperm cell performance, excessive production, and accumulation of ROS could negatively affect motility, fertility, and freezing ability of sperm (Fadl et al. 2023). In the present study, the injection of Vit E and Se significantly improved the fresh and preserved semen quality of subfertile rabbits. These findings were in line with previous studies on rabbits (Yousef et al. 2003), men (Moslemi and Tavanbakhsh 2011), chickens (Khan et al. 2012), and rams (Luo et al. 2004). This improvement in semen quality was represented by enhancing sperm motility, viability, normal morphology, concentration, and normal acrosomes.

Furthermore, sperm motility and viability are good predictors of semen quality and future fertility, and they are most extensively enhanced by treatment. The present results were in line with several previous studies in rabbits (Yousef et al. 2003), men (Moslemi and Tavanbakhsh 2011), ganders (Bas et al. 2023) rams (Luo et al. 2004; Mahmoud et al. 2013), and mice (Saddein et al. 2019). This enhancement was attributed to the synergistic effect of Vit E and Se on the antioxidant system in the rabbit sperm against ROS. Furthermore, Vit E is a non-enzymatic antioxidant that protects sperm cell membranes and organelles (mitochondria) from being attacked by ROS, it acts in two possible ways. The direct way in which Vit E inhibits the chain reaction of lipid peroxidation (Balakrishnan et al. 2013). The indirect way in which Vit E stimulates and maintains the production of scavenger antioxidant enzymes that protect cell membranes from peroxidation and reduce apoptosis (Saddein et al. 2019). In addition, Vit E has a critical role in the protection of glutathione-dependent enzymes (Van Haaften et al. 2003). Interestingly, Se is an essential element for normal non defective motility through the formation of the phospholipid–hyperoxide GSH-Px enzyme, which is a structural protein of the mitochondrial capsule in the mid-piece of mature spermatozoa (Ursini et al. 1999).

In the present investigation, G3 rabbits had higher normal sperm morphology, acrosomes, and concentration in comparison with the other two groups. These results agreed with previous studies in rabbits (Gouda et al. 2021), rams (Mahmoud et al. 2013), mice (Arjmand et al. 2023), and drakes (Mostafa et al.^2019^). This improvement was attributed to the antioxidant action of Vit E and Se against oxidative damage to testicular tissues (Bas et al. 2023). Furthermore, Vit E and Se supplements result in increasing the diameter of the seminiferous tubule and thickness of the germinal cell layer, decreasing the relative area of interstitial tissue, and consequently, increasing sperm quality and concentration (Malaniuk and Lukaszewicz 2006; Edens and Sefton 2009; Bas et al. 2023).

Testosterone secretion is highly correlated with testicular activity. In the present study, injection of Vit E and Se in subfertile rabbit bucks increased testosterone concentrations from wk2 to wk8 of treatment. These findings were in accordance with previous studies in rabbits (Gouda et al. 2021), rams (Mahmoud et al. 2013), and goat bucks (Hong et al. 2009). This increase of testosterone concentrations was attributed to the positive effect of vitamin E on the testicular tissue. Furthermore, this positive effect is represented by increasing the width of seminiferous tubules and the relative volume densities of Leydig cells, and consequently, increasing testosterone production (Hong et al. 2009).

In addition, the total antioxidant system is responsible for scavenging excessive ROS and maintaining the balance between endogenous antioxidant and ROS production (Lewis et al. 1995). Furthermore, total antioxidant competence (TAC) is a good indicator of the endogenous antioxidant system potential. In the current investigation, TAC concentrations were significantly higher in G3 from wk2 to wk8 compared to G2 and G1, respectively. These findings revealed that the injection of Vit E and Se could increase the endogenous antioxidant potential of the testes against damage by peroxidation. These results were in line with a previous study in goat bucks (Hong et al. 2010).

In the present study, G3 had higher conception and kindling rates compared to other groups (G2 and G1, respectively) when they were inseminated with either fresh or frozen-thawed treated rabbit bucks sperm suffered from infertility. These findings agreed with previous studies in men (Sabetian et al. 2021; Ener et al. 2016). This improvement in vivo reproductive performance, which was represented by increasing conception and kindling rates, may be attributed to the antioxidant action of Vit E and Se against excessive ROS and, consequently, improvement of quality parameters of rabbit spermatozoa. Moreover, vit E and Se injection in male rabbits improved the fertility rate in the New Zealand rabbit does (Zeidan et al. 2006) Which is consistent with our results in rabbit does that were inseminated with subfertile rabbit bucks injected with vit E and Se. In the meantime, several studies reported that the addition of vit E and Se to the semen extender improved the freezability of rabbit bucks’ semen in normal and under heat stress conditions (Thuwanut et al. 2013; Zhu et al. 2015). But there are no previous reports regarding the effect of vit E and Se injection effect on the freezability of rabbit bucks, especially in subfertile ones.

Interestingly, testicular morphometric measurements (weight, length, width, and volume) are good indicators for the spermatogenesis process and sperm production. In the present investigation, administration of Vit E and Se in G3 significantly increased the testicular morphometric measures compared to G2 and G1, respectively. These findings were in line with previous studies in rabbits (El-Azzazi et al. 2016; Kamel 2012), rams (Mahmoud et al. 2013) and rats (Oda et al. 2012). This increment was attributed to the positive effect of Vit E and Se on the Leydig and Sertoli cells and, consequently, increasing testosterone and androgen binding protein production. In addition, testosterone has a critical role in preserving the spermatogenesis process through increasing cell division and decreasing apoptosis (Mostafa et al. 2019). Furthermore, Vit E leads to increasing interstitial cell density and seminiferous tubules diameter (El-Azzazi et al. 2016).

## Conclusions

In conclusion, injecting Vit E (200 IU/head) and Se (0.2 mg/kg b.w) once weekly for 8weeks enhanced sperm quality, quantity, fertility and antioxidant status potential in rabbit buck sperm, as well as conferring superior protection against subfertility. However, more research is needed to study its effects on the molecular level in relation to oxidative and apoptotic genes on both levels, testes and spermatozoa.

## Data Availability

No datasets were generated or analysed during the current study.
